# Is selecting better than modifying? An investigation of arguments against germline gene editing as compared to preimplantation genetic diagnosis

**DOI:** 10.1186/s12910-019-0411-9

**Published:** 2019-11-21

**Authors:** Alix Lenia v. Hammerstein, Matthias Eggel, Nikola Biller-Andorno

**Affiliations:** Institute of Biomedical Ethics and History of Medicine, University of Zurich, Winterthurerstrasse 30, 8006 Zurich, Switzerland

**Keywords:** Bioethics; germline gene editing; preimplantation genetic diagnosis, Pediatrics, Human dignity, Physical integrity, Autonomy, Slippery slope argument, Discrimination of people living with a disability

## Abstract

**Background:**

Recent scientific advances in the field of gene editing have led to a renewed discussion on the moral acceptability of human germline modifications. Gene editing methods can be used on human embryos and gametes in order to change DNA sequences that are associated with diseases. Modifying the human germline, however, is currently illegal in many countries but has been suggested as a ‘last resort’ option in some reports. In contrast, preimplantation genetic (PGD) diagnosis is now a well-established practice within reproductive medicine. Both methods can be used to prevent children from being born with severe genetic diseases.

**Main text:**

This paper focuses on four moral concerns raised in the debate about germline gene editing (GGE) and applies them to the practice of PGD for comparison: Violation of human dignity, disrespect of the autonomy and the physical integrity of the future child, discrimination of people living with a disability and the fear of slippery slope towards immoral usage of the technology, e.g. designing children for specific third party interests. Our analysis did not reveal any fundamental differences with regard to the four concerns.

**Conclusion:**

We argue that with regard to the four arguments analyzed in this paper germline gene editing should be considered morally (at least) as acceptable as the selection of genomes on the basis of PGD. However, we also argue that any application of GGE in reproductive medicine should be put on hold until thorough and comprehensive laws have been implemented to prevent the abuse of GGE for non-medical enhancement.

## Background

The recent discovery of CrisprCas (clustered regularly interspaced short palindromic repeats - Crispr associated systems) set in motion a worldwide wave of scientific progress in the field of gene editing. CrisprCas is a comparatively cheap, efficient, precise, and easy-to-use alternative to already existing gene editing tools [1–3]. These scientific advances present major ethical concerns, especially in regard to the potential use of CrisprCas on germline cells: Spermatozoa, oocytes and their progenitors, e.g. embryonic cells in early development - cells that take part in reproduction and therefore pass on their genetic content to the next generation. At present, human germline gene editing (GGE) is prohibited by national legislation and international declarations, e.g. by the Oviedo Convention published by the Council of Europe in 1997 [4–6]. Gene editing techniques could be applied to human embryos within the context of in vitro *fertilization* (IVF) in order to modify disease associated genes and therefore interrupt the transmission of hereditary conditions. The work of Liang et al. [7] published in 2015, which uses CrisprCas on non-viable human embryos to investigate the efficacy and specificity of the method, initiated an international debate on the permissibility of such research as well as future clinical applications. While the international community was still engaged in a controversy discussion about the morality of germline gene-editing in human reproductive cells, the pot was stirred in November of 2018 when journalists covered the CrisprCas gene-edited twins born in China [8]. Opponents of GGE argue that the danger of unpredictable effects on future generations, technical difficulties compromising patient safety, as well as other serious ethical concerns outweigh potential benefits of germline gene editing [9]. Further, it is argued that with preimplantation genetic diagnosis (PGD), an effective tool for avoiding the transmission of severe hereditary diseases in assisted reproductive technology (ART) already exists, which renders the use of germline modification unnecessary for the majority of cases [9, 10]. PGD was first introduced in 1990 by a British team as a means of preventing the transmission of X-chromosomal linked disease [11]. The concept of PGD is that several embryos created via IVF treatment are analyzed for genetic anomalies associated with specific diseases, with the objective of identifying and selecting an unaffected embryo for transfer into the uterine cavity, while the remaining embryos are discarded [12]. Since the 1990s and after considerable controversy, PGD has become an established practice in Europe covered by law and national guidelines [13]. According to the Center for Genetics and Society “there is no persuasive medical reason to manipulate the human germline because inherited genetic diseases can be prevented using embryo screening techniques” [14]. Also, In 2018, the recommendation published by the European Society of Human Reproduction and Embryology (ESHRE/ ESHG) discusses adoption or gamete donation as possible alternatives to GGE, [15]. Of course, if a couple wishes to conceive a genetically related child, PGD is the only real alternative to GGE.

However, in ca. 19% of cases IVF only leads to one viable embryo [16]. In this case a parent who is a carrier of a dominant disease only has a 50% of begetting a “healthy” child. Also, for rare cases, e.g. when both parents are homozygous carriers of a recessive transmitted disease like cystic fibrosis, PGD does not represent an alternative to GGE as all produced embryos would be affected by the gene defect.

Thus, a recommendation published in 2017 by the National Academy of Science and National Academy of Medicine (NAS/NAM) concludes that clinical research on GGE in assisted reproductive technology should be considered a morally permissible option if no other alternatives exist [17]. In accordance, the recently published report by the Nuffield Council on Bioethics in 2018 concludes that GGE could be ethically acceptable if “reproductive cells that have been subject to heritable genome editing interventions are (should only be) only used for purposes that are consistent with the welfare of the future person” and if “the use of heritable genome editing interventions is (should be) consistent with social justice and solidarity so that it should not be expected to increase disadvantage, discrimination, or division in society” [18].

The ESHRE/ ESHG recommendation argues that, from a deontological perspective, GGE is morally more permissible than PGD because PGD leads to the selection between embryos instead of ‘treating’ them [15]. This begs the question whether GGE should rather be preferred over PGD, instead of being an ultima ratio option for cases of severe hereditary diseases.

To evaluate the validity of this claim, the present article analyzes and compares GGE and PGD in more detail. Four of the most prominent ethical concerns that have been raised against GGE are evaluated and compared to the practice of PGD: Violation of human dignity, disrespect of the autonomy and the physical integrity of the future child, discrimination of people living with a disability and the fear of slippery slope towards immoral usage of the technology, e.g. designing children for specific, third party interests [15, 17–20]. We selected these four concerns as they play a prominent role in public discourse and are often used as categorical arguments against GGE. By comparing GGE and PGD with a view to these arguments we want to see if PGD – as an established, legal practice in many countries – fares any better than GGE. If both technologies were comparable with regard to these arguments this would be an interesting finding given the by now widespread acceptance of PGD and the skepticism concerning GGE. We are aware that the arguments chosen represent a selection. There are many additional important issues to discuss including social justice, equality and allocation of resources within a society [10, 17, 18, 21, 22]. It would go beyond the scope of this article to address them all. Also, some of these concerns are not specific to gene editing but hold true for modern medicine in general. For instance, safety and security are very important concerns in this context. We do not want to dwell on these arguments here, for we think they would distract from ethical concerns that are more specific to germline gene editing. Therefore, for the sake of the argument, we will assume that PGD and GGE will in the near future be considered equally accessible and equally safe (while also acknowledging that at this moment in time this might not yet be the case).

The timeliness, relevance and urgency of addressing the ethical issues of germline gene editing and prenatal genetic diagnosis is highlighted by recent publications and recommendations, e.g. from the National Ethics Committee of Switzerland 2016 (NEC) [19], the National Academy of Science and the National Academy of Medicine 2017 (NAS; NAM) [17]; the Berlin Brandenburg Akademie der Wissenschaften 2015 (BBAW) [20], the Nuffield Council [18] and the background document of the European Society of Human Genetics and the European Society of Human Reproduction and Embryology 2018 (ESHG/ ESHRE) [15]. Therefore, we believe that this paper can help inform current policy discussions and may be of interest to health care professionals, in particular in the fields of reproductive medicine and pediatric care. Medical professionals play an important role in advocating children’s rights to good health care and by being involved in long term care of children living with a genetic disease they understand different aspects and impacts of certain diseases on the patient’s and the parents’ lives. Further, their opinion will guide future parents who seek advice for family planning.

## Main text

### Human dignity and the human genome

The Council of Europe emphasizes human dignity in its recommendation on Genetic Engineering in 1982 by stating that “the rights to life and to human dignity protected by Articles 2 and 3 of the European Convention on Human Rights imply the right to inherit a genetic pattern which has not been artificially changed” [23]. Building upon this, the Oviedo Convention of 1997, which serves as a legally binding treaty between its ratifying countries, prohibits the gene modification of germline cells [4]. Further, the UNESCO Declaration on the Human Genome and Human Rights in 1997 (UDHGHR) states that “the human genome underlies the fundamental unity of all members of the human family, as well as the recognition of their inherent dignity and diversity. In a symbolic sense, it is the heritage of humanity.” [24]. Human dignity has been linked to the human genome in at least two ways: The respect for the intrinsic worth of an individual human being in relation to its genome as well as the importance of the human genome for the integrity of the human species [25]. Human germline gene modification and human dignity has been discussed on both levels.

#### Risk of instrumentalization

In several recent bioethics recommendations [15, 17, 19, 20] The danger of violating human dignity by modifying the genome of a future child in order to fulfil the parental and/or societal expectations, thereby undermining the right to self-determination has been discussed. In other words, coming into existence would no longer be left to chance, but would be linked to certain - genetic - conditions.

The discussion regarding violation of human dignity often revolves around the concepts of ‘intrinsic value’ and ‘instrumentalization’. “Instrumentalization” in this context can be understood as someone (agent) using an entity (means) in a certain way (mode) for a specific purpose [26]. The concept of intrinsic value claims that all human beings have an intrinsic value that must be respected. Respect for the intrinsic value of a person demands that every person should be treated as an end in themselves and should never be reduced to their instrumental value, i.e. they should never be treated as a mere means to someone else’s end [25]. Kant formulated this in his categorical imperative as “act in such a way that you treat humanity, whether in your own person or in the person of any other, never merely as a means to an end, but always at the same time as an end. “[27].

Opponents now claim that GGE represents a risk of instrumentalization. To test the validity of this claim, we have to look at the specific context in which the technology is employed. In our example, future parents (agent) use an embryo (means) for GGE (mode) in the interest of “X” (purpose). If “X” solely means the interest of the parents to get a healthy child, then one can potentially argue that GGE in this context represents an instrumentalization since the embryo is only used as a mere means to the end of the parents (i.e. interest to get a healthy child). If “X” represents the interests of the parents for their future child and the interests of the future child to have a healthy life, e.g. if GGE is used with due respect for the child’s best interests and subject to the principle of beneficence, then it can hardly be argued that GGE is treating the future child as a mere means for the ends of the parents and as such would not represent a morally problematic form of instrumentalization.

It is difficult to see how modifying an embryo in GGE would represent a morally more problematic form of instrumentalization (i.e. treatment as a mere end) compared to discarding surplus embryos as done in PGD as long as its use is restricted to the selection of embryos based on medical characteristics such as severe hereditary monogenetic diseases.

Importantly, this is not at all to say that we oppose the generation and destruction of embryos for reproductive purposes. This is to show that it is difficult to argue against GGE on grounds of instrumentalization when comparing GGE to the morally accepted practice of PGD.

#### Integrity of the human species

In an article by Annas et al. germline genetic modification is described as a “crime against humanity” as it changes the foundation of the human species and therefore threatens human rights [28]. Bearing in mind the events leading to the Universal Declaration on Human Rights 1948 (UDHR) - World War II and the Nazi atrocities - the need to protect the human genome as a ‘consensus’ for all of humanity, without discriminating anyone on the basis of cultural or religious stigmata or mental states becomes evident. The history of eugenic practices, not only under the Nazi regime but also across the world, motivated the protection of the human genome, especially in the face of advancements in gene technology. However, the implication of this for gene modifications is unclear since there is not *one* human genome [29]. What is generally regarded as “the human genome” is a mere snapshot of evolution since all genomes are naturally undergoing constant change [30]. Although two unrelated individuals share a majority of their genes, an average human genome exhibits 4,1–5 millions variants compared to a reference genome, leading to different phenotypes including different expression of diseases [31, 32].

GGE to prevent genetically inherited diseases, would change an allele of a specific gene which is associated with a disease and would replace it with another (“healthy”) allele of the same gene. Thus, over time, no “new” genes are introduced into the gene pool, only the relative abundance of specific alleles is changed. Based on the assumption that only disease-associated genes are replaced with alleles without the specific mutation and under the assumption that the dynamic state of the human genome is included in Annas et al.’s argument on the ‘heritage of humanity’ [28], it is not evident why replacing one allele associated with a disease with another variant of the same gene would violate the integrity of the human species. Here, the Nuffield Council concludes that “there is much more to being human than the possession of a particular kind of genome” [18]. Another expression of this concern refers to ‘the naturalness’ or ‘sacredness’ of the human genome. The “naturalness” argument is based on the idea that nature is “good” and that it is wrong to intervene in nature. David Hume has argued that an ‚ought’ cannot be derived from an ‚is’ [33] and thus the foundation for the normative claim not to change nature is missing without whom one cannot derive any moral duties, responsibilities or moral guidelines for action.

Further, in today’s modern medicine it is unclear how “natural” forms of treatment are supposed to be distinguished from “unnatural” forms, e.g. most medical interventions aiming to prevent or treat a disease, such as the application of antibiotics to fight infection or resuscitation to fight death, could by the same token be considered as ‘unnatural’.

Arguments regarding the ‘sacredness’ of the human genome claim normative force by referring to the authority of god. We argue that claims regarding the authority of a divine being have little weight in secular contexts. Even if it did, it is unclear how changes to the human genome by human germline editing are fundamentally different from other (accepted and performed) actions to change or select human genes and genetic traits, e.g. selective mating (in a voluntary context, i.e. an individual choice based on perceived attractiveness of a partner or her skills [16]), epigenetic changes or PGD.

Based on this, we do not believe that the objections to GGE based on ‘naturalness’ or ‘sacredness’ hold much normative weight [17, 19, 20].

As stated, GGE could change the relative abundance of certain alleles in human populations. The same, of course, holds true for PGD. Under the assumption that no artificial or “foreign” genes are introduced through GGE, PGD and GGE are not fundamentally different with regards to their impact on the human gene pool.

### Physical integrity and autonomy of the future child

#### Physical integrity

The Child Right International Network (CRIN) refers to ‘bodily integrity’ by stating” … everyone, including children, has the right to autonomy and self-determination over their own body, and the only person with the right to make a decision about one’s body is oneself” [34]. Although not covering the complexity of the issue, the right of autonomous decisions over one’s own body and the right of self-determination are very important components in order to protect the ‘physical’ or ‘bodily’ integrity of a person [35].

In the following we will discuss the validity of the claim that GGE violates the child’s physical integrity by interfering with its genome without having the child’s consent and to what extend this can be applied to PGD [19, 20].

Opponents to GGE point to Articles 2 and 3 of the European Convention on Human Rights which imply that the future child has “the right to inherit a genetic pattern which has not been artificially changed” [23]. This argument, we think, is problematic on grounds that Gyngell et al. [16] have pointed out: “social forces have been affecting our genome for generations.[...] social and environmental influences affect gene expression through epigenetic effects, and these changes may be passed on to the next generation. “[16, 36]. Based on this, culture and parenting as well as germline gene editing affect our genome and gene expression. The difference is that the first leads to epigenetic changes (e.g. DNA methylation), whereas the second leads to changes in the actual base sequence of the DNA. However, both represent biochemical changes to the same molecule, and both lead significant phenotypic changes and as such, it is difficult to argue that one is different from the other and fundamentally different to other influences on the expression of the genome, e.g. parental decisions without the consent of the child.

The ‘non-identity problem’ was initially described by Parfit [37] and deals with the question how current actions can affect future generations, e.g. we affect the lives of future generations “by determining the kinds of social, political, economic and environmental circumstances that prevail. Our choices impact not only on what social conditions are left behind, but also who lives under those conditions. The very existence and identities of future generations depend on what choices and decisions we make. Yet if the identities of future generations depend on what we do now, then whoever exists in the future cannot claim to have been harmed by our actions when those actions turn out to be a condition of their existence.” [38].

This can be applied to PGD as follows: After a positive test result (meaning that the embryo carries the disease - associated gene mutation), parents can choose for or against one specific embryo. Therefore, the embryo will be born with its impairment or it will not come into existence as a person at all. According to the “non-identity” problem, one cannot harm someone when the alternative is non-existence. In the case of PGD, the selection of an embryo would thus not prevent nor cause harm, respectively or violate the physical autonomy or self-determination of a future child since the alternative would have been non-existence. From this, it follows that prevention of harm from a future child cannot serve as argument against or in favor of PGD.

With regard to GGE in the context of the physical integrity of the unborn child, one has to consider whether the successful correction of the gene mutation will a) change the identity of the future child, b) whether GGE is a necessary condition that an embryo is chosen for implantation, and c) whether the modification is in the best interest of the future child.

In response to the first issue, it can be argued that modifying one gene does not change the identity of a person, respectively an embryo as a person-to-be, since it changes only a very small part of its biomolecular structure, which is by nature constantly subject to change, e.g. mutations due to external stimuli such as sun light or natural mistakes in DNA duplication and repair. Of course, a severe disease can have a significant influence on a person’s life, but so do other circumstances such as education or where a child grows up. The choice of how to educate a given child will impact its life in many ways but does not lead to different children. Analogously, a life with or without a certain disease cannot be interpreted as choosing between different persons, but rather changes the course of a person’s life. Therefore, we follow the interpretation that GGE does not change the identity of an embryo as a person-to-be, since the same individual would be born - with or without a disease-causing gene.

Regarding the second, it depends whether GGE is a necessary condition for implantation. If that is the case, then GGE doesn’t represent a potential harm for the future child, for the alternative to GGE would have been ‘non-existence’.

Assuming that GGE is not a necessary condition for the parents to choose a specific embryo for implantation, e.g. parents would implant an embryo with or without GGE or irrespective of the success of GGE, then GGE can potentially harm the future child. For example, prospective parents who both carry two alleles of the gene causing cystic fibrosis may come to the decision that they will only implant the embryo if it first undergoes genetic modification. This modification might have potential harmful side-effects. Thus, to defuse the objection that GGE threatens to violate the unborn child’s physical integrity, GGE would have to be save enough that the genetic modification is allegedly in the best interest of the future child. In clinical practice, the concept of ‘informed consent’ plays a major role in protecting the physical integrity of a patient. Medical procedures may only be performed if the patient or her legal representative consents to a certain intervention after considering the relevant facts, risks and alternatives. GGE as one treatment option among others in pediatric care, the decision whether or not to proceed with it has to be taken by the parents ensuring the informed consent with regard to the best interests of their future child. Thus, applying GGE to an embryo with the best interest for the future child in mind does not per se imply a violation of its physical integrity if the resulting future child will in all likelihood not be worse off than the child from the untreated embryo would have been.

To summarize, the non-identity problem and the concern of violating the future child’s physical integrity potentially apply to GGE but not to PGD. However, when parents take the informed decision to apply GGE on an embryo in order to prevent the manifestation of a severe hereditary disease under careful consideration of risks and benefits for the child, GGE can hardly be seen as a violation of the physical integrity of the future child. While PGD can only be endorsed through reproductive autonomy - reproductive autonomy *and* acting beneficently towards the future child are potential arguments in favour of GGE. Thus, under the conditions explained above, GGE should not be seen as an infringement of the physical integrity of the future child – or at least not as a fundamentally different infringement compared to other parental (medical) decisions for their unborn or non-autonomous child, e.g. nutrition, lifestyle of pregnant mother, education, etc.

#### Autonomy

Feinberg has objected that a child has a right to an ‘open future’. He explains that children hold ‘anticipatory autonomy rights’ [39], rights that they do not have yet but will gain once they reach maturity and become capable of exercising them. One of these is the right to live an autonomous life and to make one’s own decisions e.g. concerning health care. Feinberg states that irreversible decisions should be postponed until “the child reaches maturity and is legally capable of making them himself” [39]. Thus, the right to an open future restricts what parents (and others) are allowed to do to children or the unborn child. Feinberg identifies “rights-in-trust,” as rights that „look like adult autonomy rights ... except that the child cannot very well exercise his free choice until later when he is more fully formed and capable ... rights that are to be *saved* for the child until he is an adult, but which can be violated “in advance,” so to speak, before the child is even in a position to exercise them... His right while he is still a child is to have these future options kept open until he is a fully formed self-determining adult capable of deciding among them “[39]. GGE is an irreversible decision, potentially changing major parts of a person’s life in important ways. On the account of Feinberg, the right to an open future in the discussion on GGE thus poses the question whether GGE is morally acceptable given its potential to significantly change a person’s life without having her consent.

Mills on the other hand claims that it is unclear what it means to keep options open in the context of what it means to be a “good parent” [40]: “Should our goal be to raise our children so that that they will have, as adults, as many options as possible, to give them, insofar as we can, a maximally “open” future? Or should our goal be more directive, to lead our children toward a more specifically shaped future that we ourselves endorse? “Thus, Feinberg’s theory has been criticized on the ground that it is “impossible and undesirable to try to provide children with an ‘open future’ in any meaningful sense” [41]. A lot of necessary parental decisions concerning external factors, e.g. a child’s education, religion, diet, sports, friends, neighborhood etc. are unavoidable and will have an impact on the future life of the child. Mills argues that it is not only not possible but even more importantly not desirable for parents to be “neutral” in raising their children and „steering them, however imperceptibly, toward one option rather than another “[40]. Thus, parenting always requires some (unavoidable and non-neutral) steering (making decisions for the child without its consent) which are biased by what we deem (morally) desirable or in the best interest of the child. However, the positions of Feinberg and Mills are not always necessarily mutually exclusive, especially when chosen a moderate interpretation of the right to an open future [42]. Many parenting decisions will lead to temporary closure or opening of doors. However, this is often reversible in nature and thus still maintains (according to our understanding of Feinberg) the possibility for an open future for the child. Of course, parenting will always lead to opening some doors more than others. Past experiences can never be made undone and some decisions made by parents can be re-shaped in the future while others might potentially be irreversible. But such is the nature of parenting.

Preventing a severe hereditary disease by changing the DNA sequence in an embryo potentially opens up opportunities for that individual later in life, e.g. to pursue a regular education or to become an athlete. On the other hand, the person will not experience what it means to live with the condition in question. As Gyngell et al. have pointed out, GGE to eliminate a disease would under certain circumstances rather represent an autonomy-enhancing effect (due to it’s effect on health and the possibilities for the future child) thus outweighing restrictions on autonomy “due to the presence of domination, manipulation or control” [16] of the parents. Or to rephrase, GGE to prevent severe hereditary diseases with the goal to increase the health of a future child will likely open many doors that would be closed otherwise. Likewise, it is rather „disease and disorder, not gene editing [...] that presents the greatest threat to future autonomy” [16]. Based on this, GGE cannot be rejected based on Feinberg’s account.

The concept of the right to an ‘open future’ can’t be applied to PGD: In PGD the parents choose between two embryos (existence vs non-existence) and not between two options for one embryo. This again highlights - similarly to the non-identity-problem - the conceptual difference and diverging ethical concerns between PGD and GGE, namely selection between embryos vs. therapeutic options for one embryo [10].

### Discrimination of people living with a disability

#### Social vs. medical model of disability

Central to the medical model of disability is a malfunction of the body, e.g. not being able to walk. In contrast, the social model argues that disadvantages experienced by people living with a disability are a result of the mismatch between the variability of the human body and societal norms what a body should be like, e.g. the inaccessibility of buildings or public transportation due to stairs when ambulating in a wheelchair [41, 43].

Since the introduction of prenatal testing (PNT) for malformation of the fetus or genetic aberrations followed by selective abortion in the 1970s in order to enhance reproductive choice, the disability rights movement argues that such interventions discriminate people living with a disability [44–46]. It has been argued that these tests reinforce the ‘medical model’ of disability, while leaving aside social components with the respective disability [47]. Along these lines J. Scully has pointed out that “If it is true that a significant proportion of the disadvantage of certain disabilities come from social arrangements and not the impairment *per se*, we should then be aware that prioritizing genetic interventions is choosing to tackle a socially based difficulty through biological means” [46]. GGE and PGD enforce the medical model of disability in similar ways - both methods aim to fulfil the parental wish of begetting a healthy child through a medical intervention. Thus, PGD and GGE compared to the social model can be understood as two fundamentally different approaches to the same problem.

Disability and disease are very broad terms. Disadvantages caused by one condition may be more easily socially explained - and potentially removed through changes in outer circumstances - than others. For example, whether or not being restricted in daily routine, education or work life when ambulating in a wheelchair highly depends on the infrastructure provided. Whether or not a child with trisomy 21 is included within the neighborhood and adequately supported in school heavily depends on societal attitudes and willingness to invest. In contrast, living with hereditary immunodeficiency is much harder to tackle by societal measures because bacteria and viruses can hardly be eliminated from our daily life. Thus, notwithstanding its relevance, the social model of disability can alleviate some aspects of disease burden, however, it cannot cover all of them.

#### The ‘expressivist argument’

In PGD an embryo is chosen or discarded based on a specific genetic trait. Similarly, in GGE an embryo with a specific genetic trait is subject to modification and under the assumption that the modification is successful, the embryo is chosen for implantation.

This now begs the question whether this imposes a Yes or No statement towards an embryo or future child or towards a specific genetic trait and disease, e.g. whether the choice for or against a specific genotype discriminates people living with the respective condition.

A Hasting Centre report in 1999 morally objects prenatal testing [45, 48] based on the ‘expressivist argument’ which claims that “selective abortion after prenatal diagnosis is morally problematic as it expresses negative or discriminatory attitudes, not merely about a disabling trait, but about those who carry it.” [44]. The report states that “... with discrimination more generally (...) a single trait stands in for the whole. (...) The tests send the message that there’s no need to find out about the rest” [48]. Advocates of the expressivist argument claim that PGD and GGE discriminate people living with the respective condition, as it implies that the condition is ‘not wanted’. Importantly, according to the argument, perceived discrimination is morally relevant even if discrimination was not intended.

However, there is a fundamental difference between preferring a future child not to have a specific disease and valuing life or human beings living with said disease as a life not worth living or a life less valuable. Similarly, Savulescu has challenged the general validity of the ‘expressivist argument’ by highlighting the importance of differentiating between disability and persons living with a disability. He states that “selection reduces the former, but is silent on the value of the latter” [49]. This is especially interesting in the context of medicine - In medicine, after all, it is the goal to treat diseases and most people that are suffering from a disease will undergo treatment (if available). And probably almost everybody will agree that this does not represent a discrimination of people carrying this disease.

According to Savulescu there is no moral reason to deny parents access to PGD on the basis of the ‘expressivist argument’. He points out that the individual choice of parents must not be understood as a general statement on people living with that disease. A comparison can be drawn to a case in which parents want their obese child to lose weight. The fact alone that they encourage their child to live an active life and eat healthy food to lose weight is not discriminatory towards other obese people, unless the respect, love and appreciation of the parents for the child depend on successful weight-loss. In this context, GGE should be understood as a medical option to avoid the manifestation of a severe hereditary disease. The (medical) intervention itself - the prevention of a severe hereditary disease for one specific embryo – should not be perceived as discriminatory even if the genotype of the embryo is decisive for the parental decision to use or discard of a specific embryo.

Notwithstanding that the expressivist argument and perceived discrimination of carriers of certain diseases might not stand up to ethical scrutiny, one can still discuss the moral relevance of this perceived discrimination. If it is regarded as morally relevant, then society and the medical community should be made aware of this aspect and measures could be implemented to decrease the perceived discrimination e.g. through education of the public on disability and disease and better integration of people living with a specific disease in our society. However, we do not think that PGD or GGE should be objected based on the expressivist argument.

### Slippery slope argument

The basic structure of a slippery slope argument (SSA) is that if a certain action (A) is allowed, another action (B) will necessarily follow or is very likely to follow. At the point in time when (A) is under review with regard to its moral permissibility, (B) is judged to be clearly wrong. Therefore, A must not be allowed in order to protect the prohibition of (B). The two events (A) and (B) may be linked by multiple intermediate steps [50]. The validity of a SSA - at least in a logical reasoning - depends on the likelihood of (B) and whether (A) inevitably or very likely leads to a ‘loss of control’ resulting in (B) [50]. Thus, slippery slope arguments have the structure of having „an initial, seemingly acceptable decision, (2) a dangerous outcome that is unacceptable, and (3) a process or mechanism leading from the initial decision to the dangerous outcome” [51].

Nick Agar suggested a distinction of “morally wrong” and “morally problematic” interventions. N. Agar argues that all instances of an intervention properly identified as essentially morally wrong are morally wrong (e.g. most agree that blowing up your neighbor’s car without a compelling justifying reason is always morally impermissible). However, morally problematic interventions are problematic precisely because they comprise both morally bad and morally good interventions” [52], e.g. PGD and GGE can potentially be used for gender selection, but they can also be used for the prevention and treatment of severe hereditary diseases. Based on this, GGE and PGD, should be seen as morally problematic but not morally wrong interventions because of their potential applications ranging from morally good to morally wrong [52].

Opponents of GGE formulate slippery slope arguments analogously to the above- provided SSA structure: If germline gene editing was allowed in human medicine for severe hereditary diseases (A), this would necessarily lead to violation of human dignity through eugenic use of the technology, instrumentalization of future children through non-medical enhancement and increased inequity in society through an artificial distribution of favourable biological characteristics among people living within this society (B) [15, 17, 19, 20].

#### Validity of the SSA against GGE

We will argue that the assumption that allowing GGE for medical purposes (A), e.g. treating severe hereditary diseases, inevitably or most likely leads to (B) rests on several empirical assumptions that are, prima facie, not obvious or difficult to prove.

First, for (B) to follow from (A), (B) obviously has to be scientifically feasible, i.e. it has to be scientifically possible to genetically modify strenght, eye and hair colour, height, stamina, intelligence, charisma, dexterity, agility, etc. Second, Walton coined the term “drivers” for social and political factors “driving forward of the chain of argumentation from the premises to the conclusion (the claim that the predicted disastrous outcome will occur) [...] [53]. This means that, the notion that allowance of (A) will lead to an increased moral acceptability of (B) either implies that (A) and (B) are either similar on a fundamental level or that there are obvious drivers pushing towards (B), for otherwise the causality between allowing (A) leading to (B) seems not comprehensible. Third, the loss of control caused by (A) leading to (B) rejects the possibility that regulations and laws are means to prevent sliding down the slope. We will elaborate on these assumptions below.

One potential driver is scientific developments and the probability that geneticists will be able to reliably predict what genes need to be changed in order to increase the likelihood of some ‘positive’ phenotype e.g. intelligence, athletic prowess, charisma, appearance, etc. Indeed, it may one day potentially become possible to identify and modify genes important for muscle growth, agility, dexterity, perseverance, intelligence, humor, among others. However, to this day, the knowledge regarding many of these complex traits (which are not necessarily only genetic in nature) is still limited (with eye and hair colour being exceptions). Without the scientific feasibility (A) cannot (yet) lead to (B). For the sake of the argument we will however assume that it is or soon will be scientifically feasible. In our opinion, there are, prima facie, no obvious reasons supporting the claim that only because we “could” that we actually “would” use GGE for non-medical purposes.

This claim is based on the believe that the moral acceptance for using gene editing for medical purposes would increase moral acceptance for non-medical use of GGE and eugenics. This assumptions rests on the idea that public acceptance for (A) is high, whereas (B) is perceived as clearly wrong. This view is reflected in a statement of the US National Academies stating that “with stringent oversight, heritable germline editing clinical trials could one day be permitted for serious conditions; non-heritable clinical trials should be limited to treating or preventing disease or disability at this time and that „genome editing for enhancement should not be allowed at this time “[54]. Furthermore, the assumption implies that (A) and (B) are fundamentally similar or that there are specific drivers leading to a loss of control and sliding towards (B).

The rationale behind GGE for the treatment of severe (hereditary) diseases is therapeutic, i.e. to cure a disease to generate health. The use of GGE for non-medical purposes is, as the name implies, non-therapeutic in nature, i.e. it is an enhancement and/or change of a “non-disease” phenotype, e.g. increased height or change of hair color, respectively.

Pre-implantation genetic diagnosis and plastic surgery can, in theory, also be used for non-therapeutic purposes. However, this is not seen as sufficient reason to prohibit these practices. Also, from a medical ethics point of view these two applications seem prima facie not similar but rather fundamentally different. The principle of beneficence claims that physicians have a duty to prevent harm from a patient. If GGE is understood as *therapy* and the future child as patient, then one could make an argument that physicians should act in the best interest of the future child and thus, GGE for the treatment of severe diseases could be compatible with the principle of beneficence. However, this does not hold true for GGE for non-medical purposes.

Further, we do not think that there are uncontrollable drivers pushing us down the slope. On the contrary, we think that there is at least one driver that can push us in the opposite direction. According to Walton an SSA “needs to be set in a framework of deliberation that is social, that even involves whole countries, and is highly dependent on forming policies that will set laws in place. As the technology evolves, the debates will continue, and rules will be proposed by governments formulating statutes that will be binding on the courts and will be subject to legal argumentation. What prevents the gray area from playing its part in generating a slippery slope argument is the formation of bright lines, clear rules that can tell us that we can only go so far in the sequence of actions and no further” [53]. This implies that the risk of a slippery slope depends on jurisdictions in countries and that SSAs should always take the power of laws to prevent a slippery slope from occurring into consideration.

The power of laws and regulations to prevent a slippery slope is illustrated by the following example in the Netherlands, where euthanasia has become an established part of Dutch medical practice since the 70s [55]. Euthanasia was not legalized, but ‘mercy killings’ were overlooked by Dutch prosecution. In the 90s a legislation was introduced that (although still not legalizing euthanasia) came with certain restrictions for it. Doctors now had to report cases of ‘mercy killing’ which were subject to investigation to determine if the doctors should be prosecuted. This shows how sliding on the slope may work in both directions, i.e. how moral and legal actions can prevent an uncontrolled movement from (A) to (B).

If GGE ever becomes feasible in human reproductive medicine, jurisdictions around the globe will face the difficult challenge of how to regulate it. The regulation of genetic tests including PGD is relevant here for two reasons: First, the genetic testing of an embryo will be the foundation of any application of GGE [15]. Second, it is likely that the regulation of GGE - or at least how indications are defined - will resemble the regulation of PGD in a certain country. GGE on human reproductive cells is still prohibited in many countries, e.g. Australia, Canada, Germany, Israel, Switzerland, Netherlands, whereas PGD is a widely accepted and legal practice. The regulation of PGD varies between countries around the world in mainly two aspects: How regulation is enforced (e.g. legislation vs. less enforceable guidelines) and what indications are eligible. Most European countries do have effective regulations in place [13, 56]. For example, Swiss legislation clearly restricts the use of PGD for cases of severe hereditary diseases and defines the term ‘severe hereditary disease’ through specific criteria (e.g. conditions that lead to analgesia resistant pain, reduced motoric abilities through paralysis or inability to communicate) in order to clarify which conditions are eligible for PGD [57]. However, differences within different European jurisdictions exist, e.g. some countries allow PGD for different indications, e.g. HLA typing of donor siblings (HLA compatibility of donor and recipient is important to organ or bone marrow transplantation. If a child suffers e.g. from leukaemia and no suitable bone marrow donor is found, then parents could select from a number of embryos the one embryo that would be most suitable as a donor for the older sibling) [13]. Under Belgian law, it is stated that PGD may not be used to pursue eugenic aims - further regulation is left to medical centers carrying out IVF treatments [58]. The UK has published a list of conditions approved eligible to PGD by the Human Fertilisation and Embryology Act [59]*.*

Regulation of PGD in the US and in China differ from that in Europe. The US has no federal law regulating the use of PGD (or GGE) but leaves it to the state legislation. On the whole, a liberal approach emphasizing reproductive liberty is followed, resulting in 9% of PGD usage for social sexing. Furthermore, centers exist which, upon parental request, provide PGD to select in favor of certain diseases such as dwarfism or deafness [60]. In China, an enormous increase of PGD usage was reported, highlighting differences in the societal attitude towards PGD in comparison to Western Europe. In particular, moral concerns regarding eugenic use or discrimination of disability are much less pronounced in China. Nevertheless, the Chinese government has restricted the use of PGD, which may not be used to select for non-medical features [61].

To conclude, slippery slope arguments are often based on and depend on multiple empirical claims, which are difficult to prove, however, we think that without any empirical proof, there is, prima facie, no strong reasons to accept the SSA as imperative objection to GGE. Further, attitudes and regulations regarding PGD within Europe are similar but not equal and European regulations for PGD tend to be more developed or stricter compared to the US and China. Even though we acknowledge the difficulties of guaranteeing an appropriate legal framework we argue that there is no reason to believe that a loss of control with GGE could not be prevented through appropriate laws and regulations – as the practice of PGD and the Dutch example of Euthanasia shows. We agree with Walton that “the burden of proof “regarding the likelihood of GGE for medical purposes(A) leading to the “morally catastrophic” use of GGE for eugenics (B) is on “the side of those who use the slippery slope argument against continuing with germline therapy” [53].

#### The autonomy of the parents and the right to know and not to know

Respecting patient autonomy and the “right to know”, i.e. a patient’s right to be informed about the risks and benefits of a specific treatment, has recently gained importance in medical ethics and medical practice as a fundamental ethical and legal principle [62, 63]. Recent developments in the patient-doctor relationship show a shift from a “paternalistic” model in which the doctor is allowed to withhold information towards more autonomy for the patient based on full knowledge. In this context, the “right to know” is considered of paramount importance as necessary condition for patients to make autonomous decisions. In recent years, whole genome sequencing has become available for PGD so that multiple genetic disorders can now be tested for simultaneously. Given the increasing number of choices to weigh against each other in order to find the ‘best’ option, it is important to discuss implications on decision making in modern reproductive medicine. Advocates of PGD and GGE argue that to empower future parents to take an autonomous decision, sufficient information (i.e. knowledge regarding the genetic make-up of the embryo) should be available to those that want to base their considered judgement on this kind of information.

Sandell, on the other hand, has raised concerns that this potentially represents a detrimental tendency towards a ‘hyperagency’ to master every aspect of life in general and child rearing in particular [64]. A study evaluating attitudes towards PNT has highlighted concerns about increasing social pressure upon parents. Not testing could be perceived as giving away control over the health of a future child, therefore not being a responsible parent [65]. Opponents to PGD and GGE have argued that this kind of overemphasize on autonomy might lead to a loss of the *right not to decide*. In the case of PGD and GGE, this implies that the future parent would then become morally obliged, rather than entitled, to act autonomously [66]. In light of this, it has been argued that the burden of knowledge (for patients or future parents) can potentially be unbearable and thus it was called for a *right not to know*. The rationale behind this claim is that genetic testing might provide the patient with information regarding increased risks from serious diseases without having any means to reduce these risks or to get treatment [63]. Advocates of the “right not to know” claim that knowledge of these risks are potentially too great of a burden and cause unbearable psychological stress. Along these lines, it is argued that the knowledge regarding genetic traits of an embryo can also come with the burden of knowledge and choice. It is argued that the right not to know follows from the “do no harm” principle and that autonomy leaves open the *possibility to choose* not to know and as such, is fundamentally different to a paternalistic doctor-patient model.

Together, this shows that decision making in reproductive medicine is very challenging, taking into account the emotions and uncertainty that are natural to the process of creating a family. It would be disastrous if the concept of autonomy (be it the right to know or not to know) were to be transformed into a duty to control one’s life or the future child’s life, and thus, it is of paramount importance that autonomy of future parents is secured.

PGD and GGE both represent challenging decisions regarding an equilibrium between accepting and pushing aspects of parenting. Parenting comes with the responsibility of acting in the best interest of the child without its consent. Importantly, the interpretation of what duties and responsibilities parents have is left to the autonomous parents (as long as their practice is within the limits of the law). Similarly, we believe, that PGD or GGE represent choices for the autonomous parents and as such, are not fundamentally different to widely accepted child rearing measures, as long as they are done in the best interest for the health of the future child. In light of this, arguments based on parental autonomy should not be used against the application of gene technologies in ART in order to prevent the manifestation of a severe hereditary disease but should be used to stress the importance of appropriate regulations and laws to secure the autonomy of future parents and future children.

## Conclusion

In this paper, we have discussed four arguments against the use of human GGE in the context of severe hereditary diseases. The first argument concerned the dignity of the human genome on the level of the individual and the level of the human species. We have argued that GGE to prevent a severe disease is not fundamentally different from other actions parents take without the consent of their child, e.g. as long as GGE is applied in the best interest of the future child for a healthy life and not in the interest of the parents for themselves, i.e. if the future child is an end in itself, then the intrinsic value of the future child is respected and its dignity is not violated.

In our opinion, arguments linking human dignity to the integrity of “the” human genome are weak because the human gene pool underlies constant changes and presents a great variety between its individual. It’s also unclear why and how the normative claim not to modify the human genome could be deduced from a reference to the “nature” of the genome. Moreover, in today’s medicine there is no line between “natural” and “unnatural” therapy. Also, references to the sacredness of nature based on the authority of god is not a valid reason to prohibit action in a secular world. We have also argued that in comparison to the widely accepted practice of PGD which leads to discarding surplus embryos, GGE cannot be considered a fundamentally different form of instrumentalization (assuming that either of these practices represent an instrumentalization in the first place which is a notion we do not agree with).

The second argument was related to physical integrity and the right to physical autonomy and self-determination.

The ‘non-identity problem’ described by Parfit [37] deals with the question of how current actions can affect future generations. Since one cannot argue that it’s harming someone when an alternative does not exist, we do not think that one can argue that PGD can harm the future child. GGE on the other hand can potentially harm the future child. However, we have argued that as long as GGE is considered reasonably safe and as long as GGE is applied in the best interest of the child, then GGE is not a fundamental infringement of the physical integrity or autonomy of the future child – or at least not a fundamentally different infringement compared to other parental medical decisions for their unborn child, e.g. nutrition, lifestyle of pregnant mother, etc.

J. Feinberg called for ‘anticipatory autonomy rights’ of the unborn child, such as the right to live an autonomous life and to make one’s own decisions. We argued that Mills’ idea of ‘directive and biased parenting’ is not mutually exclusive with Feinberg’s claim for a ‘maximally open future’ for the future child. In fact, we have argued that GGE is not fundamentally different to PGD or other parental decisions taken without the consent of the child in the best interest of the future child. GGE to prevent severe hereditary diseases would in fact, by bettering the health of a future child likely open more doors that would be closed otherwise. In fact, GGE is autonomy-enhancing not reducing.

Next, we turned to the argument that “selective abortion after prenatal diagnosis is morally problematic as it expresses negative or discriminatory attitudes, not merely about a disabling trait, but about those who carry it” [44]. We agreed with Savulescu who challenges the expressivist argument by highlighting the difference between disability and persons living with a disability [49]. According to Savulescu, selection implies a normative statement regarding a disease but is silent on the value of persons living with said disease [50]. We used the example of an obese child and the parents’ wish and efforts to make their child lose weight in the health interest of their child. In our opinion, the counterintuitive and clearly wrong implication of the expressivist argument would be that the parents wish and efforts in our example represent a discrimination of obese people. The expressivist argument is thus defeasible for both PGD and GGE.

The fourth argument concerned the slippery slope arguments that GGE for preventing severe hereditary diseases would lead to human enhancement and eugenics. We acknowledge, that GGE and PGD should both be considered as “morally problematic” (in an Agarian sense, because they potentially allow for morally good or morally bad interventions. However, we do not think that GGE and PGD should be considered morally wrong interventions. GGE for medical purposes is fundamentally different to GGE for non-medical applications. There are, prima facie, no reasons to believe that the moral acceptance for both is similar. Also, specific ‘drivers’ have yet to be identified that make a ‘loss of control’ and sliding down the slope towards eugenics inevitable. Importantly, as our example of euthanasia in the Netherlands has shown, laws and regulations provide a strong means not only to prevent sliding down the slope, but in fact, potentially provide a means to move up into the other direction. However, we do agree that ‘selective mentality’, hyperagency and overemphasize on autonomy in modern medicine potentially represent challenges for legalizing GGE. However, this does not provide imperative objections to PGD or GGE, but rather shows that a public and political discussion is necessary on how society defines responsible parenthood in the context of reproductive medicine and genetic tests. To prevent misuse of GGE, the practice should be put on hold until appropriate laws and regulations are put into effect. It is important that these laws promote the autonomy of the parents (be it the right to know or the right not to know) and the future child.

To summarize, we do not think that any of the discussed objections provide imperative grounds to object PGD or GGE. PGD and GGE both aim at helping parents have a healthy, genetically related child. However, the two methods differ from each other in concept and scale. With PGD parents can only select from a limited number of embryos they themselves have conceived. GGE, on the other hand, bears the potential to modify genomes to have specific wanted traits. Following the arguments provided in this paper, the claim that the genetic modification of an embryo in order to prevent the manifestation of a severe hereditary disease is at least as morally acceptable as PGD, as postulated by the ESHRE/ ESHG, can be considered valid under the following conditions: Only gene variants that already exist within the human gene pool are used and GGE is restricted to promoting the *child’s* interest in good health in cases of severe hereditary diseases.

However, this paper is not a plea to rush towards clinical use of GGE. Our conclusion is limited to the arguments discussed above. As pointed out in the introduction, other important moral concerns (e.g. social justice, distribution of scarce resources, intergenerational relationships [67]) will need to be taken into consideration, too. It is important to mention again that we set all safety and technical issues aside for the sake of the argument. Naturally, prior to any clinical implementation of GGE, addressing these concerns including a harm-benefit-analysis is inevitable.

## Data Availability

Not applicable.

## Abbreviations

BBAW: Berlin Brandenburg Akademie der Wissenschaften
CRIN: The Child International Network
Crispr Cas: Clustered regularly interspaced short palindromic repeats - Crispr associated systems
ESHRE/ESHG: European Society of Human Reproduction and Embryology
GGE: Germline gene editing
IVF: In-vitro-fertilization
NAS/ NAM: National Academy of Science and National Academy of Medicine
NEC: National Ethics Committee of Switzerland
PGD: Preimplantation genetic diagnosis
PNT: Prenatal testing
SSA: Slippery slope argument
UDHGHR: UNESCO Declaration on the Human Genome and Human Rights
UDHR: Universal Declaration on Human Rights
